# Renal Dysfunction and Tubulopathy Induced by High-Dose Tenofovir Disoproxil Fumarate in C57BL/6 Mice

**DOI:** 10.3390/healthcare8040417

**Published:** 2020-10-21

**Authors:** Eungyeong Jang, Jong Kil Lee, Kyung-Soo Inn, Eun Kyoung Chung, Kyung-Tae Lee, Jang-Hoon Lee

**Affiliations:** 1 Department of Internal Medicine, College of Korean Medicine, Kyung Hee University, Seoul 02447, Korea; obliviona79@naver.com; 2 Department of Internal Medicine, Kyung Hee University Korean Medicine Hospital, Seoul 02447, Korea; 3 Department of Fundamental Pharmaceutical Science, Graduate School, Kyung Hee University, Seoul 02447, Korea; jklee3984@gmail.com; 4 Department of Life and Nanopharmaceutical Sciences, Graduate School, Kyung Hee University, Seoul 02447, Korea; innks@khu.ac.kr; 5 Department of Pharmacy, College of Pharmacy, Kyung Hee University, Seoul 02447, Korea; cekchung@khu.ac.kr; 6 Department of Pharmaceutical Biochemistry, College of Pharmacy, Kyung Hee University, Seoul 02447, Korea

**Keywords:** tenofovir disoproxil fumarate, nephrotoxicity, renal tubulopathy

## Abstract

Tenofovir disoproxil fumarate (TDF) is the most preferred antiretroviral medicine in treating human immunodeficiency virus (HIV) and hepatitis B virus (HBV) infections. Recent clinical trials have reported conflicting results on renal toxicity and safety in TDF-treated patients, but reference animal studies, testing high-doses of TDF for renal toxicity, are scarce. In this preclinical study, we investigated whether daily oral TDF administration (200, 500, or 800 mg/kg/d, *p*.*o*.) for four weeks induces renal insufficiency in C57BL/6 mice, by evaluating changes in body weight, urine micro-total protein, urinary microalbumin, serum blood urea nitrogen (BUN), and creatinine levels, along with histological examination of kidney samples. In the G3 group (TDF 800 mg/kg/d, *p*.*o*.), three mice died on the 17th, 23rd and 26th days, and overall, significant increases in urinary and serum levels were observed after two weeks of TDF treatment. In addition, the proportion of pyknotic epithelial cells and acidophilic cytoplasm in renal tubules was also increased after two weeks, and congestion and hemorrhage were observed in renal tubules after three weeks. Taken together, high-dose TDF treatment of 800 mg/kg/d might lead to renal tubular damage and dysfunction, great enough to cause death in mice, even after a short period of one to two weeks.

## 1. Introduction

Since the US Food and Drug Administration (FDA) approval of tenofovir disoproxil fumarate (TDF) as a favorable antiretroviral agent for human immunodeficiency virus (HIV) in 2001 and subsequently for hepatitis B virus (HBV) in 2008, TDF, an analogue of adenosine 5′-monophosphate, has been widely used to preferentially treat viral infectious diseases such as HIV and HBV [1]. As befits its therapeutic reputation as a first-line therapy for acquired immune deficiency syndrome (AIDS) or chronic hepatitis B (CHB), it has proven to have more potent antiviral effects, excellent tolerability, convenient administration (one pill per a day with or without food), and lower toxicity [2] than adefovir or lamivudine [3] in treating co-infection as well as HIV and HBV independently. 

As for renal toxicity induced by TDF, however, there have been numerous conflicting results from previous preclinical and clinical studies [1,2,4,5,6]. In multiple studies, clinically significant nephrotoxic effects were observed at a negligible level of 0.5~1% following analysis of post-marketing safety data [4], and data from a cohort study [7] along with data from a systematic review and meta-analysis [5]. Although relatively uncommon, ever since TDF-induced Fanconi syndrome and renal failure was found in a patient with HIV for the first time in 2002 [8], more reports have been made related to the renal toxicity of TDF [6]. For pre-clinical studies before drug approval, different species like male Sprague Dawley rats, hemizygous HIV-1 transgenic (TG) mice, woodchucks, rhesus monkeys, and rhesus macaques were tested for renal toxicity with TDF at doses ranging from 0.125~600 mg/kg/d [9,10,11,12,13,14]. Nevertheless, most studies have been incomplete in providing detailed information regarding renal function changes, renal safety and toxicity according to dosing over time. Additionally, high-dose TDF-treated in vivo studies are required to predict clinically severe renal problems because an appropriate dose of TDF, which is considered safe, could unpredictably cause detrimental effects on the kidneys in patients with renal insufficiency. However, there have been no murine studies with administration of 800 mg/kg/d of TDF, which is about 13-fold of the human use dose [15]. 

In order to investigate its renal toxicity under high-dose treatment, we analyzed changing patterns of urine micro-total protein, urinary microalbumin, blood urea nitrogen (BUN), creatinine, body weight, and kidney histopathological characteristics during four weeks per oral (*p.o*.) administration of TDF 200, 500, or 800 mg/kg/d.

## 2. Materials and Methods 

### 2.1. Materials

**Figure 1:**
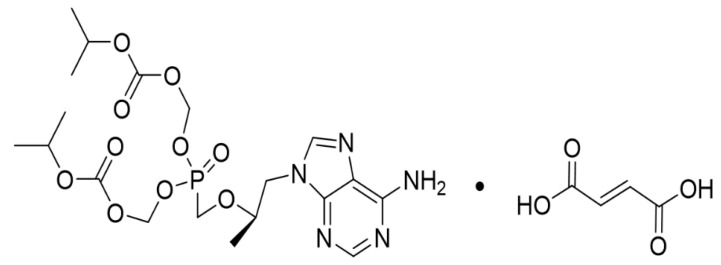
Chemical structure of tenofovir disoproxil fumarate (TDF).

TDF was obtained from LAURUS Labs (Visakhapatnam, India) and its purity was recorded as 99.6% by assay by HPLC (Figure 1). Water content of it was 0.4% and the melting range was measured from 115 to 116 °C. Zoletil 50 (VIRBAC, Carros, France) and xylazine (Rompun^®^, Bayer AG, Leverkusen, Germany) were purchased from VSParm Co., Ltd. (Seoul, Korea). Hematoxylin and Eosin (H&E, BBC, Mount Vernon, VA, USA) was obtained from Dreambio (Seoul, Korea).

### 2.2. Animals and Experimental Design

C57BL/6NCrljOri male mice (7 weeks) which were purchased from Orient Bio (Jungwon-gu, Seongnam, Gyeonggi-do, Korea) and maintained under constant conditions (temperature, 23 ± 3 °C; humidity, 55 ± 15%; the number of ventilation, 10–20 times/h; lightning time, 12 h; intensity of illumination, 150~300 Lux). At 24 h before the experiment, only water was provided. All procedures were conducted in accordance with the Animal Experimentation Policy of Korea guidelines and approved by the ethical committee for the Korea Animal Medical Science Institute (16-KE-166). The total 48 mice which were regarded as healthy were assigned randomly into one control group and three treatment groups (*n* = 12/group) according to the ranking of body weight, which make the body weight of each group similar levels: the vehicle-treated control group (G0), the TDF 200 mg/kg/d-treated group (G1), the TDF 500 mg/kg/d-treated group (G2), and the TDF 800 mg/kg/d-treated group (G3). TDF was administered orally at 200, 500, or 800 mg/kg daily for four weeks. 

### 2.3. Evaluation of Body Weight

The relevant mice were checked for body weight on the TDF administration beginning day, and then were examined once per week.

### 2.4. Sample Collection

For each group with three mice, an autopsy was carried out 4 times over four weeks. The relevant mice were anesthetized each week on an autopsy day as previously arranged, and then their blood was gathered through the vena cava in the abdomen. After blood collection, the mice were euthenized by Zoletil 50 (VIRBAC, Carros, France, 5 mg/kg) and xylazine (Rompun^®^, Bayer AG, Leverkusen, Germany, 2.5 mg/kg) intraperitoneally using 1 mL syringe equipped 26 gauge needlle, after which excised kidneys were fixed in 10% neutral buffered formalin solution. Blood was injected into a vacutainer tube, and then coagulated for about 15 min at a room temperature. Afterwards, it was centrifuged for 10 min at 3000 rpm to separate serum. Serum was stored in the deep freezer set to less than −70 °C until analysis. The guidelines of KNOTUS Co., Ltd. (Incheon, Korea) were implemented in our experiment, and data from moribund state mice were contained in each group. Although the day of collecting blood in the moribund state did not exactly match the other mice’s, it was collected and analyzed according to each week. Therefore, the sample size can be regarded as a total of 48 mice.

### 2.5. Evaluation of Micro-Total Protein and Microalbumin from Urine

The day before sacrifice for each of the four weeks, the living mice were placed in metabolic cages. After collecting the urine, micro-total protein and microalbumin, they were analyzed by a blood chemistry analyzer (7020 Hitachi, Tokyo, Japan).

### 2.6. Evaluation of BUN and Creatinine from Serum

Serum BUN and creatinine from the blood sample obtained when the mice were sacrificed were analyzed by a blood chemistry analyzer (7020 Hitachi).

### 2.7. Histopathological Analysis of the Kidney

Fixed kidney tissues were manufactured for an experimental specimen after going through general tissue treatment processes such as roughing, dehydration, embedding in paraffin, and cutting. To implement the histopathological analysis, these tissues were stained with Hematoxylin and Eosin (H&E), and then observed to examine the change of histopathological characteristics with the aid of light microscope (Olympus BX53, Tokyo, Japan).

### 2.8. Statistical Analysis

The biochemical results and body weights in our study were analyzed by an ANOVA and the least significant difference (LSD) multiple comparison tests performed by PASW^®^ Statistics 18 (SPSS Ltd., QB, Hong Kong, China)**.** Values at *p* < 0.05 were considered to be statistically significant.

## 3. Results

### 3.1. Effects of TDF on Body Weight Changes

Body weight gains in the G0 and G1 groups gradually increased every week until necropsy day. In contrast, the proportional weight loss per week was markedly observed in the G2 and G3 groups. In the week 4 necropsy group, body weights in week 4 increased by 4% and 7% in the G0 and G1 group compared to the baseline data in week 0, respectively, whereas there was a 7% decrease from week 0 until week 4 in the G2 group. In particular, the body weight of the G3 group in week 4 showed significant weight loss compared to the control group in week 4 as well as initial weight of the G3 group in week 0 (*p* < 0.05, Table 1).

### 3.2. Effects of TDF on Urine Micro-Total Protein and Microalbumin

**Figure 2:**
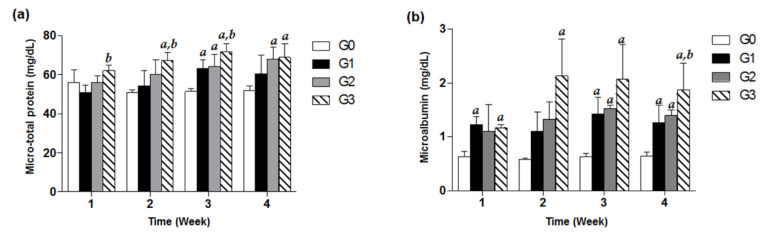
Effects of TDF on urine micro-total protein and microalbumin in mice. Forty-eight mice were divided into four groups, and each group (G0–G3) contained 12 mice. Each of the three treatment groups consisted of 12 mice: G1 group (TDF 200 mg/kg/d, *p.o*.), G2 group (TDF 500 mg/kg/d, *p.o*.), and G3 group (TDF 800 mg/kg/d, *p.o*.). Urinary micro-total protein (**a**) and microalbumin (**b**) levels of each group measured every week for four weeks were presented as a bar graph. The data are expressed as mean ± S.D. The comparison among the groups was performed by the ANOVA test followed by the LSD test. *a*: *p* < 0.05 compared with the G0 group, *b*: *p* < 0.05 compared with the G1 group.

Treatment with TDF (200, 500, 800 mg/kg/d) exhibited increasing values in urine micro-total protein and microalbumin with respect to the control group in a time-dependent manner for 4 weeks. Significant increases in urine microalbumin compared with control in the G1 and G3 groups were observed from only week 1, suggesting that urine microalbumin levels of study groups are more susceptible to TDF treatment than urine micro-total protein values. In case of the G2 group, there was a significant increase in microalbumin from week 3. Of note, the microalbumin data for the G3 group showed about a 3-fold significant increase in comparison with the control G0 group for four weeks of TDF treatment (*p* < 0.05, Figure 2). 

### 3.3. Effects of TDF on Serum BUN and Creatinine

**Figure 3:**
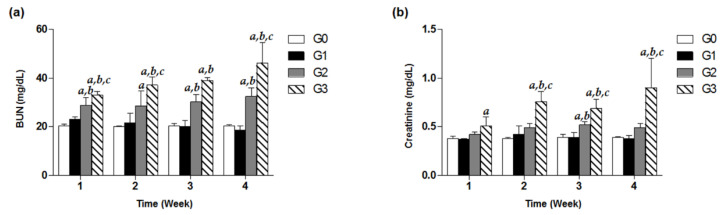
Effects of TDF on serum BUN and creatinine levels in mice. Forty-eight mice were divided into four groups, and each group (G0–G3) contained 12 mice. Mice were treated with various doses of TDF (200, 500, or 800 mg/kg/d, *p.o*.) for four weeks. Serum BUN (**a**) and creatinine (**b**) levels of each group measured every week for four weeks are presented as a bar graph. The data are expressed as mean ± S.D. The comparison among the groups was performed by the ANOVA test followed by the LSD test. *a*: *p* < 0.05 compared with the G0 group, *b*: *p* < 0.05 compared with the G1 group, *c*: *p* < 0.05 compared with the G3 group.

To investigate decline in renal function induced by high-dose TDF treatment, we observed the kidney function parameters including serum BUN and creatinine of G0–G3 group for four weeks. In the G3 group under the treatment of 800 mg/kg/d for only one week, a significant increase in BUN was recorded by approximately 1.5-fold compared to that of the control, and even time-dependently increased throughout the treatment days (Figure 3a). Similarly, the levels of creatinine in a high-dose G3 group from week 2 to week 4 were observed to be significantly different from the estimates in the G0, G1, and G2 groups (Figure 3b). Meanwhile, the G2 group showed significantly higher levels of BUN in the serum analysis over four weeks, compared with the control group (*p* < 0.05, Figure 3a). In contrast, there were no remarkable increases in BUN and creatinine levels in the G1 group for four weeks (Figure 3). 

### 3.4. Effects of TDF on Histological Manifestations of Renal Tissues

**Figure 4:**
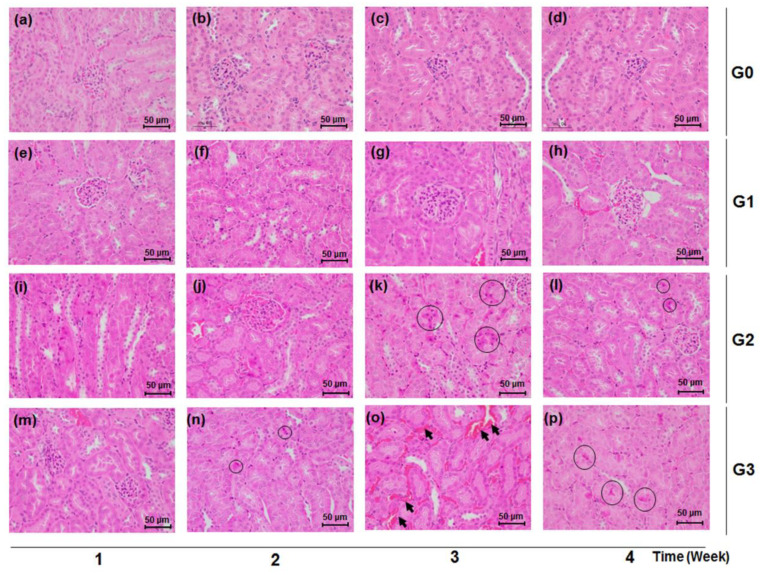
Effects of TDF on renal tissue histology in mice. Mice were sacrificed each week over a four week period after TDF administration. Renal tissues were stained with Hematoxylin and Eosin (H&E) (×400). Black circles indicate renal tubular necrotic (pyknotic) cells and black arrows point to congestion and hemorrhage in renal tubules, respectively. Scale bar is 50 μm. (**a**) G0 group, week 1; (**b**) G0 group, week 2; (**c**) G0 group, week 3; (**d**) G0 group, week 4; (**e**) G1 group, week 1; (**f**) G1 group, week 2; (**g**) G1 group, week 3; (**h**) G1 group, week 4; (**i**) G2 group, week 1; (**j**) G2 group, week 2; (**k**) G2 group, week 3; (**l**) G2 group, week 4; (**m**) G3 group, week 1; (**n**) G3 group, week 2; (**o**) G3 group, week 3; (**p**) G3 group, week 4.

Consistent with the findings in the urinary and serum biochemical data, the kidney tissues of mice treated with TDF of 500 mg/kg/d and 800 mg/kg/d showed histological damage compared with the normal renal tissues of the control group. The TDF-induced histological renal toxic changes were characterized by the appearance of necrotic cells, congestion, and hemorrhage in renal tubules (Figure 4). There exists no significant histological manifestation from the renal tissue of the control (G0) and low-dose (G1) groups (Figure 4a–h), while renal tubular pyknotic cells were observed in the kidney tissues of the G3 and G2 groups from week 2 and week 3, respectively (Figure 4k,l,n–p). Tubular congestion and hemorrhage were markedly shown in the G3 group at week 3 (Figure 4o). 

## 4. Discussion

There have been reports that a variety of kidney diseases such as membranoproliferative glomerulonephritis (MPGN), membranous glomerulonephritis, and polyarteritis nodosa (PAN) are closely associated with HBV infection [16,17], and kidney problems are considered important comorbidities among patients infected with retroviruses [18]. For these HBV or HIV patients with kidney dysfunction, the first-line recommended medicine is TDF which is primarily excreted through the renal tubules and thereby the kidney plays a pivotal role in its metabolic process. In particular, symptomatic or severe renal impairment, like a rapid decline in the estimated glomerular filtration rate, proteinuria, high creatinine, and chronic kidney disease was aggravated during TDF exposure, while its discontinuation improved kidney-related problems [19]. Despite this potential to stimulate renal toxicity in HBV or HIV patients, a precise molecular mechanism or a specific target related to the renal toxicity of TDF has yet to be elucidated [11]. Therefore, more detailed and specific evidence regarding TDF renal toxicity is required to augment previous experimental studies and anticipate its potential toxicity in a real-life clinical situation. 

Our results suggest for the first time that oral prescription of TDF could induce significant changes in biological markers and histological indications related to kidney toxicity according to dosage and duration for a treatment period of four weeks. For biological markers, both albuminuria and proteinuria are indicative of renal dysfunction in chronic kidney diseases [20], and BUN and creatinine levels also correlate with kidney pathology as indirect but important markers of function [21]. 

Regarding the administration dose of TDF, 200 mg/kg/d, 500 mg/kg/d and 800 mg/kg/d doses were selected in this study. It has been reported that the TDF *C*max and AUC were dose-proportional following single and multiple doses of TDF in the dose range of 75–600 mg in healthy fasted or HIV-infected adults. In addition, the multiple-dose pharmacokinetics of TDF were evaluated after seven consecutive days of administration of TDF 75, 150, 300 or 600 mg once daily with food to 38 HIV-infected patients. Median *C*max values were dose proportional and the median steady-state pharmacokinetic parameters were dose-linear across all dose groups. TDF is eliminated unchanged in the urine by a combination of glomerular filtration and proximal tubular secretion [22]. Subsequently the drug is secreted to the tubular lumen by the apical membrane transporters multidrug resistance protein (MRP)-4 and MRP-2. A relationship between TDF exposure and kidney tubular dysfunction (KTD) was found, in that the TDF plasma trough concentration was higher in patients with KTD than in the rest. This dose-dependent nephrotoxic effect further supports an involvement of the blood TDF concentration in KTD [23]. Based on these previous experiments, we suggest that our data are in agreement with prior findings in which high dose TDF administration is correlated with higher plasma levels which may lead directly to a greater accumulation of TDF in the renal tubular cells and, consequently, to kidney toxicity.

In our present study, three mice in the G3 group died from day 17 to day 26 out of the total of 48 C57BL/6NCrljOri mice during the four weeks of TDF-treatment: mouse 43, 46 and 47 died from TDF high-dose on the 17th, 23th and 26th days. This high mortality rate of mice in our study suggested that a TDF dose of 800 mg/kg/d for four weeks, which is about 13 times that of the recommended human dosage, was considerably lethal in the mouse model. A study longer than four weeks would also be useful, as many patients have to take daily TDF for longer periods of time. 

In the case of the G1 group, meaningful indications in renal histological findings associated with renal toxicity were not observed after four weeks of dosing. The body weights in each week necroscopy group: week 1, week 2, week 3 and week 4 of the G1 group time-dependently increased until the date of necropsy, as compared with the respective baseline values (Table 1). Neither BUN nor creatinine levels in the serum significantly increased to the levels suggestive of renal toxicity (Figure 3). Although urinary microalbumin significantly increased in the G1 group after 1, 3 and 4 week treatment (Figure 2b), oral administration of TDF at a dose of 200 mg/kg/d for four weeks proved to be generally safe in mice in other results. This was consistent with previous in vivo outcomes in which no side effects showing mitochondrial injury were seen in SD rats treated with TDF (300 mg/kg/d, *p.o.*) for four weeks [12]. 

Oral administration of TDF at a dose of 500 mg/kg/d led to abnormal changes in kidney function parameters, indicating the beginnings of nephrotoxicity. The G2 group exhibited significant weight loss compared to the G0 group at week 4 after treatment, suggesting that 500 mg/kg of TDF per day was harmful to mice (*p* < 0.05, Table 1). As shown in Figure 3a, the G2 group showed a significant rise to 28.86 ± 2.99 mg/dL of BUN in the serum on the first week, considerably higher than the 20.25 ± 0.86 mg/dL of the G0 control group. After three weeks of being on 500 mg/kg/d, *p.o*. TDF, the microalbumin level in urine and serum creatinine had also conspicuously increased in a time-dependent manner, and pyknotic epithelial cells began to accumulate in the proximal and distal convoluted tubules of the renal cortex, as compared with no change in the G0 and G1 groups (Figure 2, Figure 3 and Figure 4). As for micro-total protein levels, they steadily increased over time for four weeks, and significant changes (*p* < 0.05) were observed from the third week, compared with the control group (Figure 2a). 

Of interest were the findings in our study for the G3 group on TDF 800 mg/kg/d, a dosage that was comparable to approximately 13 times of the human equivalent therapeutic recommended dose based on calculations using a conversion factor across species [15]. The G3 group time-dependently showed considerable increases in urinary micro-total protein and microalbumin levels, as well as serum BUN and creatinine levels. In particular, at four weeks of TDF treatment, the level of kidney-function markers such as microalbumin, BUN, and creatinine had more than doubled in comparison with those of the control group (Figure 2 and Figure 3). Additionally, unlike a recent study showing no significant changes (*p* < 0.05) in creatinine levels in a TDF 600 mg/kg/day treated group at five weeks [14], we could observe a considerably higher level of creatinine in this G3 group at four weeks (Figure 3b). These unusual increases in BUN and creatinine levels were consistently shown in the moribund states in the G3 group, confirming that a sufficiently high dosage of TDF, over 10-fold that of the standard dosage, could impair renal function and structure in patients as well as study animals (Figure 3). Of note, for kidney histological samples from the test mice, there were no remarkable changes in the G0 and G1 groups over the four weeks, but there had been a severe dissolution of cell nucleus and a cytoplasmic acidophilic change noted in the proximal and distal tubules of the renal cortex from the G3 group and G2 group since week 2 and week 3 treatment, respectively (Figure 4k,l,n–p). In addition, the section of kidney samples from the G3 group after three weeks of TDF treatment showed marked renal tubular congestion and hemorrhagic damage (Figure 4o). Renal histological damage, resulting from drug-induced acute kidney injury, presents different findings according to the drug. Certain drugs such as ciprofloxacin and ampicillin are closely associated with crystal accumulation in renal tissue [24], and drugs like rifampin and vancomycin can induce tubular cell toxicity [25]. TDF-induced renal toxicity commonly results in tubulo-interstitial disease and several cases reporting it have been reported in clinical settings [26]. A human renal biopsy specimen of a patient receiving TDF showed severe tubular necrosis with distinct interstitial fibro-edema [27], and its histologic examination indicating tubulopathy might be similar to the marked tubular congestion from the results in our study. However, considering the following limitations of our study, caution should be exercised when interpreting and applying our study findings. In this study, nephrotoxicity was exclusively assessed without evaluating possible toxic effects on other organs. Because fatal events occurred in our current study, the observed nephrotoxic events might potentially result from systemic lethal damage. In addition, the dose tested in our current study was equivalent to approximately a 13-times higher dose than that commonly used in humans. We acknowledge the maximum dose evaluated in our study (i.e., 800 mg/kg) might be excessively high based on the currently approved TDF dose in humans (max. of 300 mg/day). However, clinical drug interaction studies of TDF typically suggested approximately a 30% increase in tenofovir systemic exposures (max.: 98% increase [28]). Based on these studies, the estimated maximum dose to be tested in our study was 800 mg/kg to account for potential drug interaction consequences of TDF. Furthermore, our maximum dose was within the TDF dose range in a previous toxicokinetic study where TDF toxicity was assessed in the dose range of 50 to 1000 mg/kg [29], showing dose-dependent toxicities in mice. Considering that the highest dose investigated in this study was higher than the maximum dose approved in humans, additional studies are necessary to increase the robustness of our study findings because the Animal-to-Human Uncertainty Factor in toxicological studies is commonly set at approximately 10. Future studies are required to examine kidney-specific toxicity induced by high-dose TDF by evaluating the effect on other organs and to investigate the potential toxic effects of TDF over a broad range of doses including doses lower than 800 mg/kg.

## 5. Conclusions

Consequently, the in-vivo results in the present study may demonstrate that high-dose TDF treatment for four weeks induces tubular histological damages as well as abnormal kidney dysfunctions, such as significant increases in micro-total protein, microalbumin, BUN, and creatinine in C57BL/6 mice. Based on these observations of kidney structure and function with TDF dosing, further studies on TDF-induced renal toxicity, the effects of higher doses, longer durations of treatment, and also possible interactions with other drugs are required. This is necessary in order to reduce the possibility of severe kidney problems in patients on TDF for long periods and who are also taking other medications. 

## Figures and Tables

**Table 1:** Body weight changes in the week 4 necropsy group administered with TDF for four consecutive weeks.

Group	Week 0			Week 1			Week 2			Week 3			Week 4		
G0	19.82	±	1.25	20.05	±	1.36	20.21	±	1.45	20.40	±	1.49	20.63	±	1.50
G1	19.86	±	0.91	20.39	±	0.71	20.95	±	0.86	21.15	±	0.94	21.33	±	0.97
G2	19.82	±	0.88	19.39	±	0.87	19.03	±	0.70	18.74	±	0.82	18.41	±	1.08 ^#^
G3	19.81	±	0.88	19.37	±	0.84	18.76	±	1.30	17.71	±	1.77	16.66	±	2.26 *^,#^

The body weight data (g) are expressed as mean ± S.D. The comparison among the groups was performed by the ANOVA test followed by the least significant difference (LSD) test. *: *p* < 0.05 compared with the week 0 group, ^#^: *p* < 0.05 compared with the G0 group.
